# Supplementary material for: RUFY1 binds Arl8b and mediates endosome-to-TGN CI-M6PR retrieval for cargo sorting to lysosomes
Source: J Cell Biol. 2022 Oct 25;222(1):e202108001. doi: 10.1083/jcb.202108001 (PMC9597352; doi:10.1083/jcb.202108001)
Supplement: Table S2 — is a list of DNA constructs and primer sequences used in this study. [file JCB_202108001_TableS2.docx]

**Supplementary Table II:**

**1. List of DNA constructs used in this study.**

| **Plasmid Name** | **Description** | **Source** |
| --- | --- | --- |
| ***Mammalian expression constructs:*** | | |
| pcDNA3.1(-) | Mammalian expression vector | Invitrogen |
| pcDNA3.1(-)-Arl8b (WT)-HA | Full-length human Arl8b with C-terminal HA tag cloned in pcDNA3.1(-) vector | Described previously (Marwaha et al., 2017) |
| pcDNA3.1(-)-Arl8b (Q75L)-HA | Full-length human Arl8b with Q75L mutation and C-terminal HA tag cloned in pcDNA3.1(-) vector | Described previously  (Marwaha et al., 2017) |
| pcDNA3.1(-)-Arl8b (T34N)-HA | Full-length human Arl8b with T34N mutation and C-terminal HA tag cloned in pcDNA3.1(-) vector | Described previously  (Marwaha et al., 2017) |
| pcDNA3.1(+)-RUFY1-FLAG | Full-length human RUFY1 (variant 1; isoform a; 708 a.a. in length, also known as Rabip4’) with C-terminal FLAG tag cloned in pcDNA3.1(+) vector | GenScript (USA) |
| pcDNA3.1(-)-Rabip4-FLAG | Full-length human Rabip4 (isoform b; 600 a.a. in length) with C-terminal FLAG tag cloned in pcDNA3.1(-) vector | This study |
| pcDNA3.1(-)-RUFY1 (RR→A)-FLAG | Full-length human RUFY1 with point mutations at aa positions Arg206 and Arg208 to Ala and C-terminal FLAG tag cloned in pcDNA3.1(-) vector | This study |
| pcDNA3.1(-)-RUFY1 (ΔRUN)-FLAG | C-terminal FLAG-tagged-human RUFY1 lacking 1-271 a.a. cloned in pcDNA3.1(-) vector | This study |
| pEGFP-N1-RUFY1 | Full-length human RUFY1 cloned in pEGFP-N1 vector | This study |
| pEGFP-C1-Rab14 | Full-length human Rab14 cloned in pEGFP-C1 vector | Gift from Prof. Isabelle Coppens (Johns Hopkins University, USA) |
| pCDH-CMV-EF1-PURO-TAP- RUFY1 | N-terminal TAP-tagged full-length-human RUFY1 cloned in pCDH-CMV-MCS-EF1-Puro vector | This study |
| pEGFP-N1-RUFY1 (WT) Rescue construct | Full-length human RUFY1 rescue construct against RUFY1 siRNA #1 cloned in pEGFP-N1 vector | This study |
| pEGFP-N1-RUFY1 (ΔCC2) Rescue construct | Human RUFY1 rescue construct against RUFY1 siRNA # 1 with internal deletion of 400-500 a.a. cloned in pEGFP-N1 vector | This study |
| pcDNA3.1(-)-2x-FKBP-GFP | 2x-FKBP12 with C-terminal GFP-tag cloned in pcDNA3.1(-) vector | Described previously (Dwivedi et al., 2019) |
| pcDNA3.1(-)-2x-FKBP-GFP-RUFY1 (FL) | Full-length human RUFY1 with N-terminal 2x-FKBP-GFP cloned in pcDNA3.1(-) vector | This study |
| pcDNA3.1(-)-2x-FKBP-GFP-RUFY1 (1-500 a.a.) | Human RUFY1 (1-500 a.a.) with N-terminal 2x-FKBP-GFP cloned in pcDNA3.1(-) vector | This study |
| pcDNA3.1(-)-2x-FKBP-GFP-RUFY1 (1-400 a.a.) | Human RUFY1 (1-400 a.a.) with N-terminal 2x-FKBP-GFP cloned in pcDNA3.1(-) vector | This study |
| pcDNA3.1(-)-2x-FKBP-GFP-RUFY1 (1-300 a.a.) | Human RUFY1 (1-300 a.a.) with N-terminal 2x-FKBP-GFP cloned in pcDNA3.1(-) vector | This study |
| pcDNA3.1(-)-2x-FKBP-GFP-RUFY1 (ΔCC1) | Human RUFY1 lacking 300-400 a.a. with N-terminal 2x-FKBP-GFP and a C-terminal FLAG tag cloned in pcDNA3.1(-) vector | This study |
| pcDNA3.1(-)-2x-FKBP-GFP-RUFY1 (ΔCC2) | Human RUFY1 lacking 400-500 a.a. with N-terminal 2xFKBP-GFP and a C-terminal FLAG tag cloned in pcDNA3.1(-) vector | This study |
| pcDNA3.1(-)-2x-FKBP-GFP-RUFY1 (ΔCC1+ΔCC2) | Human RUFY1 lacking 300-500 a.a. with N-terminal 2xFKBP-GFP and a C-terminal FLAG tag cloned in pcDNA3.1(-) vector | This study |
| pcDNA3.1(-)-2x-FKBP-GFP-RUFY1 (ΔRUN) | Human RUFY1 lacking 1-271 a.a. with N-terminal 2x-FKBP-GFP cloned in pcDNA3.1(-) vector | This study |
| Mito-FRB | Mito (Tom70p) fused to FRB | Gift from Prof. Martin Lowe (The University of Manchester, UK) |
| pDsRed-C1-Rab14 | Full-length human Rab14 cloned in pDsRed-C1 vector | This study |
| Str-KDEL-IRES-SBP-mCherry-CTSZ | RUSH construct: the hook protein (Streptavidin-KDEL) and cargo protein (Streptavidin-binding protein-mCherry-Cathepsin Z) are cloned in the same MCS, the two ORFs are linked by an internal-ribosome entry site (IRES) element of pIRESneo3 vector | Gift from Prof. Junjie Hu (Institute of Biophysics, Chinese Academy of Sciences, China) |
| LAMP1-GFP | LAMP1 fused to GFP | Gift from Prof. Steve Caplan (University of Nebraska Medical Center) |
| pEGFP-C1-SNX1 | Full-length mouse SNX1 cloned in pEGFP-C1 vector | Gift from Prof. Paul Gleeson Lab (University of Melbourne, Australia) |
| CD8α-CI-MPR-pIRES Neo2 | A chimera of ectodomain of CD8 and cytoplasmic tail of the bovine CI-M6PR cloned in pIRESneo2 vector | Gift from Prof. Matthew Seaman (Cambridge Institute for Medical Research, UK) |
| pcDNA3.1(+)-Arl8b (Rescue construct) | Full-length human Arl8b (without any tag) rescue construct against Arl8b siRNA cloned in pcDNA3.1(+) vector | Described previously (Marwaha et al., 2017) |
| Arl8b (Q75L)-BirA^*^-HA-MAO | Human Arl8b lacking first 17 amino acids and with Q75L mutation cloned in JB93 vector (Addgene plasmid # 128904) by replacing Rab7a (QL) cassette | This study |
| Rab14 (Q70L)-BirA^*^-HA-MAO | Human Rab14 lacking last three amino acids (Δcys form) with Q70L mutation cloned in JB93 vector (Addgene plasmid # 128904) by replacing Rab7a (QL) cassette | This study |
| Rab4a (Q72L)-BirA^*^-HA-MAO | Human Rab4a lacking last three amino acids (Δcys form) with Q72L mutation cloned in JB93 vector (Addgene plasmid # 128904) by replacing Rab7a (QL) cassette | This study |
| Rab5a (Q79L)-BirA^*^-HA-MAO | Human Rab5a lacking last three amino acids (Δcys form) with Q79L mutation cloned in JB28 vector | Gift from Prof. Sean Munro (MRC Laboratory of Molecular Biology, UK; Addgene plasmid # 128900) |
| pcDNA3.1(−)-FLAG-SKIP | N-terminal FLAG-tagged full-length human SKIP cloned into the pcDNA3.1(-) vector | Described previously (Marwaha et al., 2017) |
| mCherry-Rabip4 | N-terminal mCherry-tagged human Rabip4 cloned in pcDNA3 vector | Gift from Prof. Hye-Won Shin (Kyoto University, Japan) |
| pEYFP-Golgi | A fusion protein consisting of enhanced yellow fluorescent protein (EYFP) and a sequence encoding the N-terminal 81 amino acids of human beta 1,4-galactosyltransferase | Clontech |
| pcDNA3.1(-)-RUFY3-FLAG | C-terminal FLAG-tagged full-length human RUFY3 (variant 1; 620 a.a. in length) cloned in pcDNA3.1(-) vector | Described previously (Gaurav et al., 2022) |
| ***Bacterial expression constructs:*** | | |
| pGEX-4T3 vector | Bacterial protein expression vector for making N-terminal GST proteins | GE HealthCare |
| pGEX-4T3-Arl8b | Full-length human Arl8b with N-terminal GST tag cloned in pGEX-4T3 vector | Described previously  (Marwaha et al., 2017) |
| pET15b (+)-Arl8b (WT) | Full-length human Arl8b with N-terminal His tag cloned in pET15b (+) vector | Described previously (Marwaha et al., 2017) |
| pET15b(+)-Arl8b (Q75L) | Full-length human Arl8b (Q75L) with N-terminal His tag cloned in pET15b (+) vector | This study |
| pET15b(+)-Arl8b (T34N) | Full-length human Arl8b (T34N) with N-terminal His tag cloned in pET15b (+) vector | This study |
| pGEX-4T3-RUFY1 (RUN) | Human RUFY1(1-302 a.a.) cloned in pGEX-4T3 vector | This study |
| pRSF2-His-Rab7 | Rab7 with N-terminal His tag cloned into the pRSF2 vector | Described previously (Marwaha et al., 2017) |
| pGEX-4T3-RUFY1 | Full-length human RUFY1 (longer isoform; 708 a.a.) with N-terminal GST tag cloned in pGEX-4T3 vector | This study |
| pMAL-C2X vector | Bacterial protein expression vector for making N-terminal maltose-binding protein (MBP)-tagged proteins | (Described previously Dwivedi et al.,2019) |
| pMAL-C2X-Rab14 | Human Rab14 lacking last three amino acids (Δcys form) with N-terminal MBP tag cloned in pMAL-C2X vector | This study |
| pGEX-5X1-LIC1 (WT) | Human LIC1 (389-523 a.a.) cloned into pGEX-5X1 vector | (Described previously Dwivedi et al.,2019) |
| pGEX-5X1-LIC1 (FFAA) | Human LIC1 (389-523 a.a.) with point mutations at aa positions Phe447 and Phe448 to Ala cloned into pGEX-5X1 vector | This study |
| ***Yeast two-hybrid constructs*** | | |
| pGADT7 vector | GAL4-activation domain yeast two-hybrid vector | Takara Bio Inc. |
| pGADT7-RUFY1 (WT) | Full length human RUFY1 cloned into pGADT7 vector | This study |
| pGADT7-RUFY1 (NΔRUN) | Human RUFY1 lacking 147-270 a.a. cloned into pGADT7 vector | This study |
| pGADT7-RUFY1 (ΔFYVE) | Human RUFY1 (1-594 a.a.) cloned into pGADT7 vector | This study |
| pGBKT7 vector | GAL4-DNA binding domain yeast two-hybrid vector | Takara Bio Inc. |
| pGBKT7-Rab14 (Q70L) | Rab14 (Q70L) lacking last three amino acids (Δcys form) cloned in pGBKT7 vector | Gift from Prof. Coen Kuijil (Amsterdam University Medical Center, Amsterdam) |

**2. List of the primers used in this study.**

Fp: Forward primer; Rp: Reverse primer; SDM: Site directed Mutagenesis; SOE: Splicing by overlap extension

| **Construct** | **Primers sequences used for making the designated construct** |
| --- | --- |
| pcDNA3.1(-)-Rabip4-FLAG | Fp NheI: 5’-CTAGCTAGCATGATGGAGGAGCGTGCCAAC-3’ |
|  | Rp BamHI: 5’-CGGGATCCTCACTTGTCGTCATCGTCTTTGTAGTCCA  TGGAGGCCGTGGAGGAGCAGC-3’ |
| pcDNA3.1(-)-RUFY1 (RR→A)-FLAG | SDM Fp: 5’-AAGACAGCTGTGGGAGCAGGCGCAGCGTGGCTTTATCT-3’ |
|  | SDM Rp: 5’-AGATAAAGCCACGCTGCGCCTGCTCCCACAGCTGTCTT-3’ |
| pcDNA3.1(-)-RUFY1 (ΔRUN)-FLAG | Fp EcoRI: 5’-CGGAATTCATGGACTTGGATTCTCAGGTTGGAG-3’ |
|  | Rp BamHI: 5’-CGGGATCCTCACTTGTCGTCATCGTCTTTGTAGTCCA  TGGAGGCCGTGGAGGAGCAGC-3’ |
| pEGFP-N1-RUFY1 (WT) Rescue construct | SOE Fp: 5’-TCCAGAAGCATCCGACATTGCCACAAGTGTCAGAAATC  TTC-3’ |
|  | SOE Rp: 5’-GAAGATTTCTGACACTTGTGGCAATGTCGGATGCTTCT  GGA-3’ |
| pEGFP-N1-RUFY1 | Fp EcoRI: 5’-CGGAATTCATGGCCGACCGGGAAGGCG-3’ |
|  | Rp BamHI: 5’-CGGGATCCCCGGAGGCCGTGGAGGAGCAGC-3’ |
| pEGFP-N1-RUFY1 (ΔCC2) Rescue construct | SOE Fp: 5’-TCCAGAAGCATCCGACATTGCCACAAGTGTCAGAA  ATCTTC-3’ |
|  | SOE Rp: 5’-GAAGATTTCTGACACTTGTGGCAATGTCGGATGCT  TCTGGA-3’ |
| pDsRed-C1-Rab14 | Fp XhoI: 5’- CCGCTCGAGCTATGGCAACTGCACCATACAAC-3’ |
|  | Rp EcoRI: 5’-CCGGAATTCCTAGCAGCCACAGCCTTCTCT-3’ |
| pCDH-CMV-EF1-PURO-TAP-RUFY1 | Fp NheI: 5’-CTAGCTAGCATGAAGCGACGATGGAAAAAGAA-3’ |
|  | Rp EcoRI: 5’-CGGAATTCTCAGGAGGCCGTGGAGGAGC-3’ |
| pcDNA3.1(-)-2x-FKBP-GFP-RUFY1 (FL) | Fp XhoI: 5’-CCGCTCGAGATGGCCGACCGGGAAGG-3’ |
|  | Rp BamHI: 5’-CGGGATCCTCAGGAGGCCGTGGAGGAG-3’ |
| pcDNA3.1(-)-2x-FKBP-GFP-RUFY1 (1-500 aa) | Fp SDM: 5’-GTTATGTCCAGCATGAAACAATGAGAAGAAAGGTTGCAG  CACTCGG-3’ |
|  | Rp SDM: 5’-CCGAGTGCTGCAACCTTTCTTCTCATTGTTTCATGCTGGAC  ATAAC-3’ |
| pcDNA3.1(-)-2x-FKBP-GFP-RUFY1 (1-400 aa) | Fp SDM: 5’-CAAGGTCTGGATGAAATGTACTGAGATGTGTGGAAGCAGC  TAAAAG-3’ |
|  | Rp SDM :5’-CTTTTAGCTGCTTCCACACATCTCAGTACATTTCATCCAGA  CCTTG-3’ |
| pcDNA3.1(-)-2x-FKBP-GFP-RUFY1 (1-300 aa) | Fp SDM: 5’-GTGGCAAGGAGCATGAAAGATGAACTGATGTCCTTGATCA  AA-3’ |
|  | Rp SDM: 5’-TTTGATCAAGGACATCAGTTCATCTTTCATGCTCCTTGCCA  C-3’ |
| pcDNA3.1(-)-2x-FKBP-GFP-RUFY1 (ΔCC1) | Fp SDM: 5’- GGTGGCAAGGAGCATGAAAGAAGTGATGTGTGGAAGCAG  CTA-3’ |
|  | Rp SDM: 5’- TAGCTGCTTCCACACATCACTTCTTTCATGCTCCTTGCCAC  C-3’ |
| pcDNA3.1(-)-2x-FKBP-GFP-RUFY1 (ΔCC2) | Fp SDM: 5’-GGCAAGGTCTGGATGAAATGTACATGGAAGAAAGGTTGCA  GCAC-3’ |
|  | Rp SDM: 5’- GTGCTGCAACCTTTCTTCCATGTACATTTCATCCAGACCTT  GCC-3’ |
| pcDNA3.1(-)-2x-FKBP-GFP-RUFY1 (ΔCC1+ΔCC2) | Fp SDM: 5’-GGATCTTGATGGTGGCAAGGAGCATGAAAGAATGGAAGAA  AGGTTGCAGCACTCGGAGCGGGCG-3’ |
|  | Rp SDM: 5’**-**CGCCCGCTCCGAGTGCTGCAACCTTTCTTCCATTCTTTCATG  CTCCTTGCCACCATCAAGATCC-3’ |
| pcDNA3.1(-)-2x-FKBP-GFP-RUFY1 (ΔRUN) | Fp XhoI: 5’-CCGCTCGAGATGGACTTGGATTCTCAGGTTGGAGTAATA-3’ |
|  | Rp BamHI: 5’-CGGGATCCTCAGGAGGCCGTGGAGGAG-3’ |
| Rab14 (Q70L)-BirA^*^-HA-MAO | Fp SDM: 5’-CTGCAGATTTGGGATACGGCAGGACTGGAGCGATTTAGG  GCTGTTACACGG -3’ |
|  | Rp SDM:5’-CCGTGTAACAGCCCTAAATCGCTCCAGTCCTGCCGTATCC  CAAATCTGCAG -3’ |
| Rab4a (Q72L)-BirA^*^-HA-MAO | Fp SDM: 5’-GTAAAGTTACAAATATGGGATACAGCAGGACTAGAACGA  TTCAGGTCCGTGACGAGAAGT-3’ |
|  | Rp SDM**:** 5’-ACTTCTCGTCACGGACCTGAATCGTTCTAGTCCTGCTGTA  TCCCATATTTGTAACTTTAC **-**3’ |
| Arl8b (Q75L)-BirA^*^-HA-MAO | Fp SDM: 5’-GGGACATAGGAGGACTACCCCGATTTCGAAGC-3’ |
|  | Rp SDM: 5’-GCTTCGAAATCGGGGTAGTCCTCCTATGTCCC-3’ |
| pGEX-4T3-RUFY1 (RUN) | Fp EcoRI: 5’-CGGAATTCCATGGCCGACCGGGAAGGCGGCT-3’ |
|  | Rp XhoI : 5’-CCGCTCGAGTCAAGTAATTCTTTCATGCTCCTTGC-3’ |
| pGEX-4T3-RUFY1 | Fp EcoRI :5’-CGGAATTCCATGGCCGACCGGGAAGGCGGCT-3’ |
|  | Rp EcoRI: 5’-CGGAATTCTCAGGAGGCCGTGGAGGAGCAG-3’ |
| pET15b(+)-Arl8b (Q75L) | Fp SDM: 5’-GGGACATAGGAGGACTACCCCGATTTCGAAGC-3’ |
|  | Rp SDM: 5’-GCTTCGAAATCGGGGTAGTCCTCCTATGTCCC-3’ |
| pET15b(+)-Arl8b (T34N) | Fp SDM: 5’-CAGTACTCGGGCAAGAACACCTTCGTCAATGTC-3’ |
|  | Rp SDM:5’-GACATTGACGAAGGTGTTCTTGCCCGAGTACTG-3’ |
| pMAL-C2X-Rab14 | Fp BamHI: 5’-CGCGGATCCATGGCAACTGCACCATACAAC-3’ |
|  | Rp SalI: 5’-ACGCGTCGACCTAGCCTTCTCTCTGGGGTTGGGG-3’ |
| pGEX-5X1-LIC1 (FFAA) | Fp SDM: 5’-AAGGCGTTCTGGCAAATGCCGCCAACAGTTTGTTGAGTA  A-3’ |
|  | Rp SDM: 5’-TTACTCAACAAACTGTTGGCGGCATTTGCCAGAACGCCT  T-3’ |
| pGEX-5X1-LIC1 (WT) | Fp BamHI: 5’-CGGGATCCTTACTGCAGCTGGAAGGCCTGTGG-3’ |
|  | Rp XhoI: 5’-CGGCTCGAGCTAAGAAGCTTCTCCTTCCGTAGGAG-3’ |
| pGADT7-RUFY1 (WT) | Fp EcoRI: 5’-CGGAATTCATGGCCGACCGGGAAGGCG-3’ |
|  | Rp BamHI: 5’-CGGGATCCTCAGGAGGCCGTGGAGGAG-3’ |
| pGADT7-RUFY1 (ΔFYVE) | Fp EcoRI: 5’-CGGAATTCATGGCCGACCGGGAAGGCG-3’ |
|  | Rp BamHI: 5’-CGGGATCCTCAGTCCTGAAGCTCCCGCAACTCC-3’ |
| pGADT7-RUFY1 (NΔRUN) | Fp SOE: 5’-GCGGACCATGCCCCCTTGCAGGAAGACTTGGATTCTCAGG  T-3’ |
|  | Rp SOE: 5’-ACCTGAGAATCCAAGTCTTCCTGCAAGGGGGCATGGTCCG  C-3’ |
