# Supplementary material for: RUFY1 binds Arl8b and mediates endosome-to-TGN CI-M6PR retrieval for cargo sorting to lysosomes
Source: J Cell Biol. 2022 Oct 25;222(1):e202108001. doi: 10.1083/jcb.202108001 (PMC9597352; doi:10.1083/jcb.202108001)
Supplement: Table S3 — is a list of antibodies used in this study. [file JCB_202108001_TableS3.docx]

**Supplementary Table III**: List of antibodies used in this study.

(WB: Western Blotting; IF: Immunofluorescence; IP: Immunoprecipitation)

| **Antibody** | **Source** | **Identifier** |
| --- | --- | --- |
| Mouse anti-RUFY1  (IF-1:100)  (WB-1:1000; Fig. 4E, Fig. S2L, Fig. S5A) | Santa Cruz Biotechnology | sc-398740 |
| Rabbit anti-RUFY1  (WB-1:1000; Fig. 1B, Fig. 1E, Fig. 4G, Fig. 4I, Fig. 8A, Fig. S1A)  (IP-2µg; Fig. 8A, Fig. S5A) | Abcam | ab241080 |
| Mouse anti-CI-M6PR  (IF-1:500) | Abcam | ab2733 |
| Rabbit anti-CI-M6PR  (IF-1:500) | Abcam | ab124767 |
| Rabbit anti-SNX1  (IF-1:200) | Proteintech | 10304-1-AP |
| Mouse anti-SNX1  (IF-1:200) | BD Biosciences | 611482 |
| Mouse anti-EEA1  (IF-1:200) | BD Biosciences | 610457 |
| Rabbit anti-EEA1  (IF-1:200) | Cell Signaling Technology | 3288 |
| Mouse anti-LAMP1  (IF-1:1000)  (WB-1:1000) | BD Biosciences | 555798 |
| Rabbit anti-LAMP1  (IF-1:2500) | Abcam | ab24170 |
| Rabbit anti-Rab14  (IF-1:150; Fig. S4M-O, Fig. S5G-I)  (WB-1:3000; Fig. 4G) | Sigma-Aldrich | R0565 |
| Rabbit anti-Rab14  (IF-1:100; Fig. 2B, Fig. 4A-B, Fig. S1D, Fig. S2C) | Abcam | ab28639 |
| Mouse anti-Rab14  (WB-1:1000; Fig. S3K)  (IP-2µg; Fig. S4G) | Santa Cruz Biotechnology | sc-271401 |
| Mouse anti-Vps35  (IF-1:100) | Santa Cruz Biotechnology | sc-374372 |
| Mouse anti-CD8  (IF-1:500) | BD Biosciences | 555631 |
| Mouse anti-Tom20  (IF-1:500) | Santa Cruz Biotechnology | sc-17764 |
| Rabbit anti-Cathepsin D  (IF-1:500)  (WB-1:2000) | Abcam | ab75852 |
| Mouse anti-Rab7  (IF-1:100) | Santa Cruz Biotechnology | sc-376362 |
| Mouse anti-HA  (IF-1:500)  (WB-1:3000) | BioLegend | MMS-101P |
| Rabbit anti-HA  (IF-1:250)  (WB-1:3000) | Sigma-Aldrich | H6908 |
| Mouse anti-FLAG  (IF-1:500)  (WB-1:3000) | Sigma-Aldrich | F1804 |
| Rabbit anti-FLAG  (IF-1:500)  (WB-1:3000) | Sigma-Aldrich | F7425 |
| Mouse anti-GFP  (WB-1:5000) | Santa Cruz Biotechnology | sc-9996 |
| Mouse anti-His  (WB-1:5000) | Sigma-Aldrich | SAB1305538 |
| Rabbit anti-Arl8b  (WB-1:1000; Fig. S2F) | Cell Signaling Technology | 56085 |
| Mouse anti-α-tubulin  (WB-1:10000) | Sigma-Aldrich | T9026 |
| Rabbit anti-Vps26  (IF-1:250) | Abcam | ab23892 |
| Mouse anti-dynein intermediate chain  (WB-1:5000) | BioLegend | 904901 |
| Mouse anti-p150^glued^  (WB-1:3000) | BD Biosciences | 610474 |
| Rabbit anti-transferrin receptor  (WB-1:3000) | Abcam | ab84036 |
| Mouse anti-GAPDH  (WB-1:3000) | Santa Cruz Biotechnology | sc-166574 |
| Rabbit anti-Giantin  (IF-1:4000) | Abcam | ab24586 |
| Mouse anti-EGFR  (IF-1:500) | Invitrogen | MA5-13269 |
| Mouse anti-Rab8  (WB-1:3000)  (IP-2µg) | BD Biosciences | 610844 |
| Rabbit anti-RSK1  (WB-1:1000)  (IP-2µg) | Cell Signaling Technology | 9355 |
| Rabbit anti-Arl8  (IF-1:50; Fig. 1F, Fig. 4C)  (WB-1:1000; Fig. 1E) | Custom-made | Previously described  (Garg et al., 2011; Marwaha et al., 2017) |
| Rabbit anti-PLEKHM1  (WB-1:3000) | Gift from P. Odgren (University of Massachusetts Medical School, Worcester, MA) |  |
| Mouse anti-Arl8-conjugated agarose  beads  (IP-30 μL slurry) | Santa Cruz Biotechnology | sc-398635 AC |
| HRP-conjugated goat anti-rabbit IgG  (WB-1:5000) | Jackson ImmunoResearch | 111-035-144 |
| HRP-conjugated goat anti-mouse IgG  (WB-1:5000) | Jackson ImmunoResearch | 115-035-166 |
| Alexa-Fluor 488-conjugated  goat anti-rabbit IgG  (IF-1:500) | Thermo Fisher Scientific | A11034 |
| Alexa-Fluor 568-conjugated  goat anti-rabbit IgG  (IF-1:500) | Thermo Fisher Scientific | A11036 |
| Alexa-Fluor 488-conjugated  goat anti-mouse IgG  (IF-1:500) | Thermo Fisher Scientific | A11029 |
| Alexa-Fluor 568-conjugated  goat anti-mouse IgG  (IF-1:500) | Thermo Fisher Scientific | A11031 |
| Alexa-Fluor 647-conjugated  goat anti-mouse IgG  (IF-1:500) | Thermo Fisher Scientific | A21235 |
| Alexa-Fluor 647-conjugated  goat anti-rabbit IgG  (IF-1:500) | Thermo Fisher Scientific | A21245 |
