# Supplementary figures and images for: RUFY1 binds Arl8b and mediates endosome-to-TGN CI-M6PR retrieval for cargo sorting to lysosomes
Source: J Cell Biol. 2022 Oct 25;222(1):e202108001. doi: 10.1083/jcb.202108001 (PMC9597352; doi:10.1083/jcb.202108001)

Source Data Main Fig.1

Fig. 1B

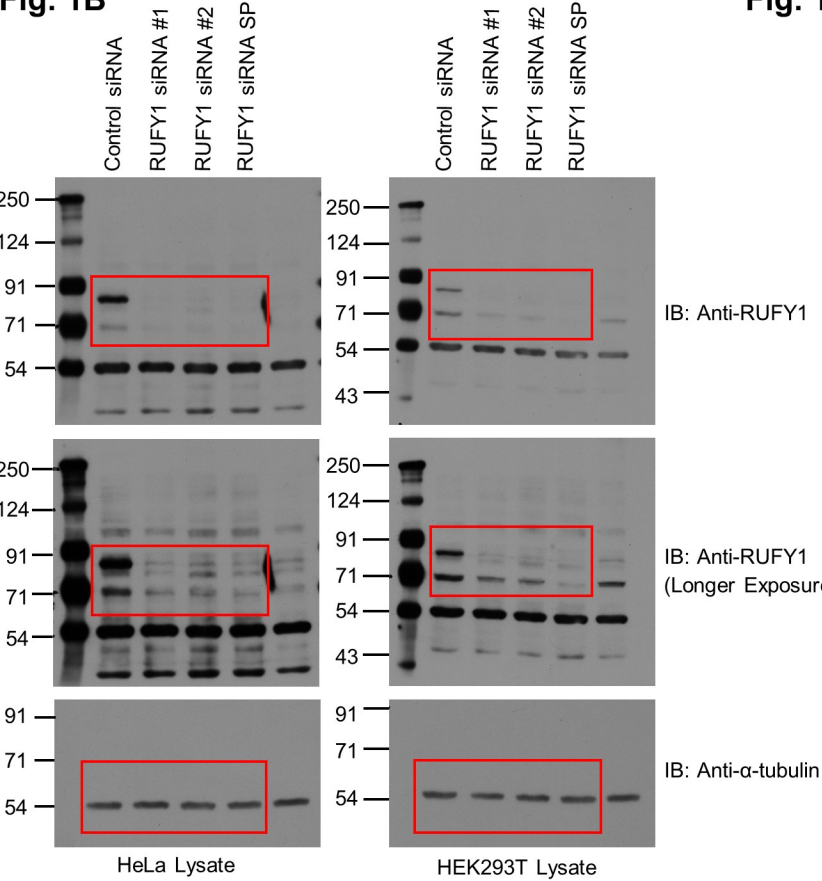

Fig. 1C

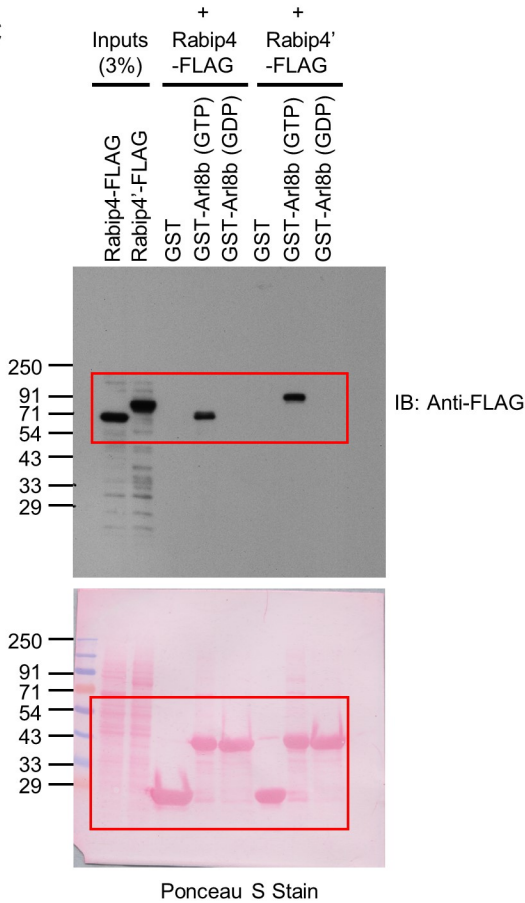

Fig. 1D

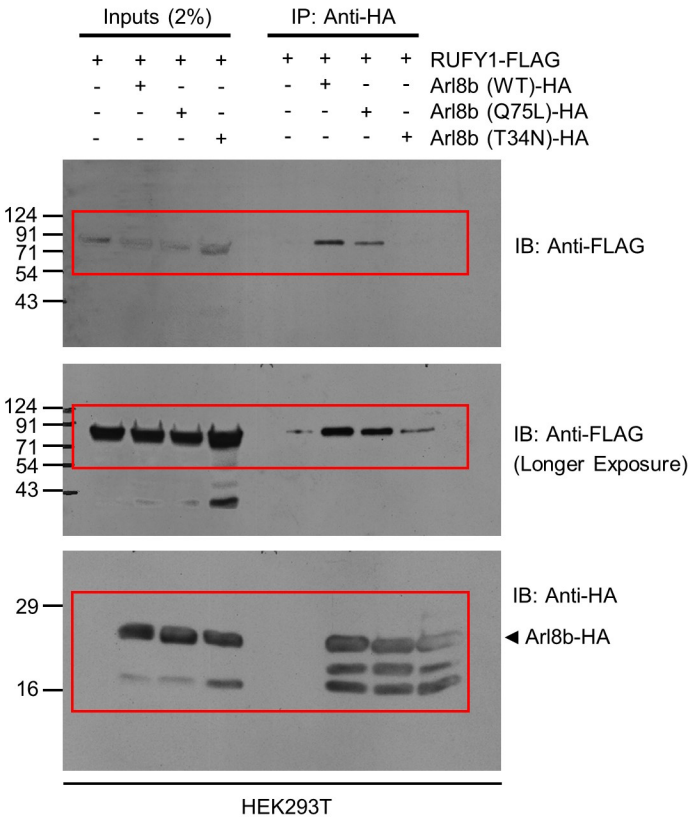

Fig. 1E

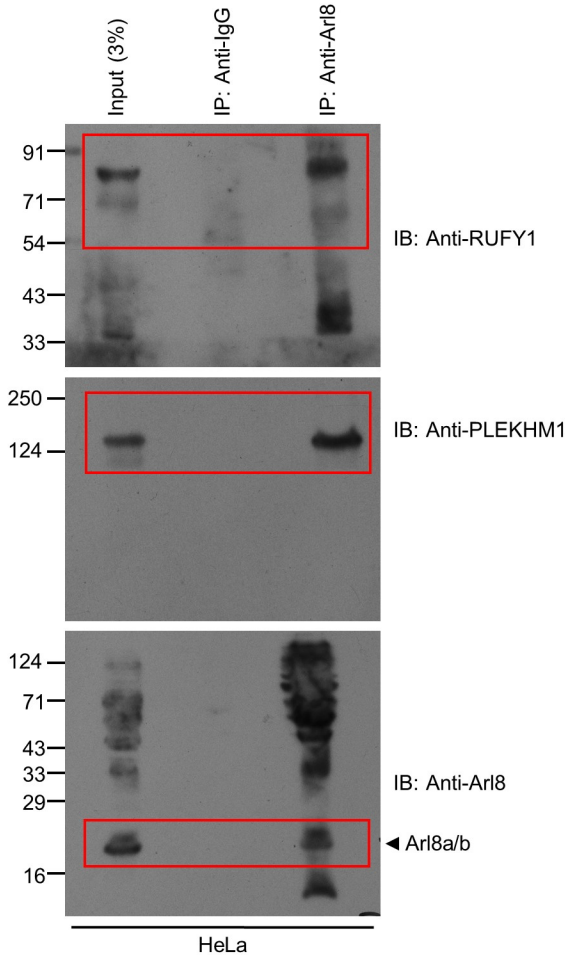

Supplement: SourceData F1 — is the source file for Fig. 1. [file JCB_202108001_SourceDataF1.pdf]

# Source Data Main Fig.3

Fig. 3B

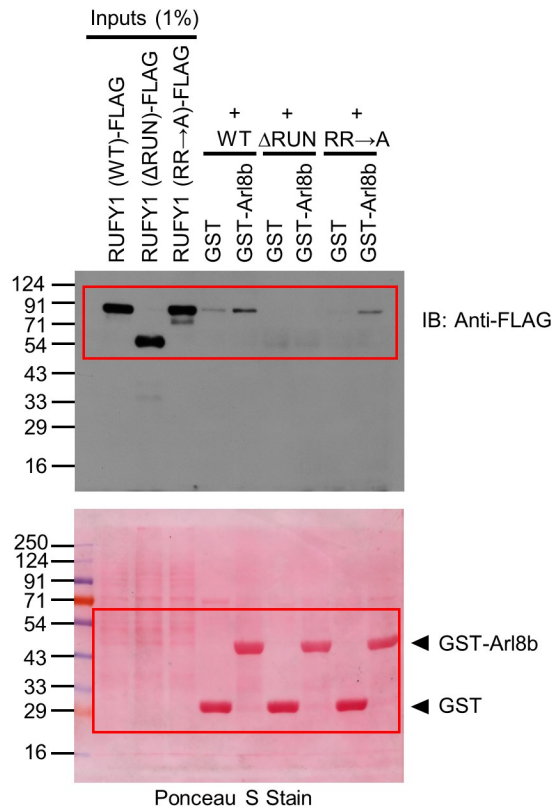

Fig. 3D

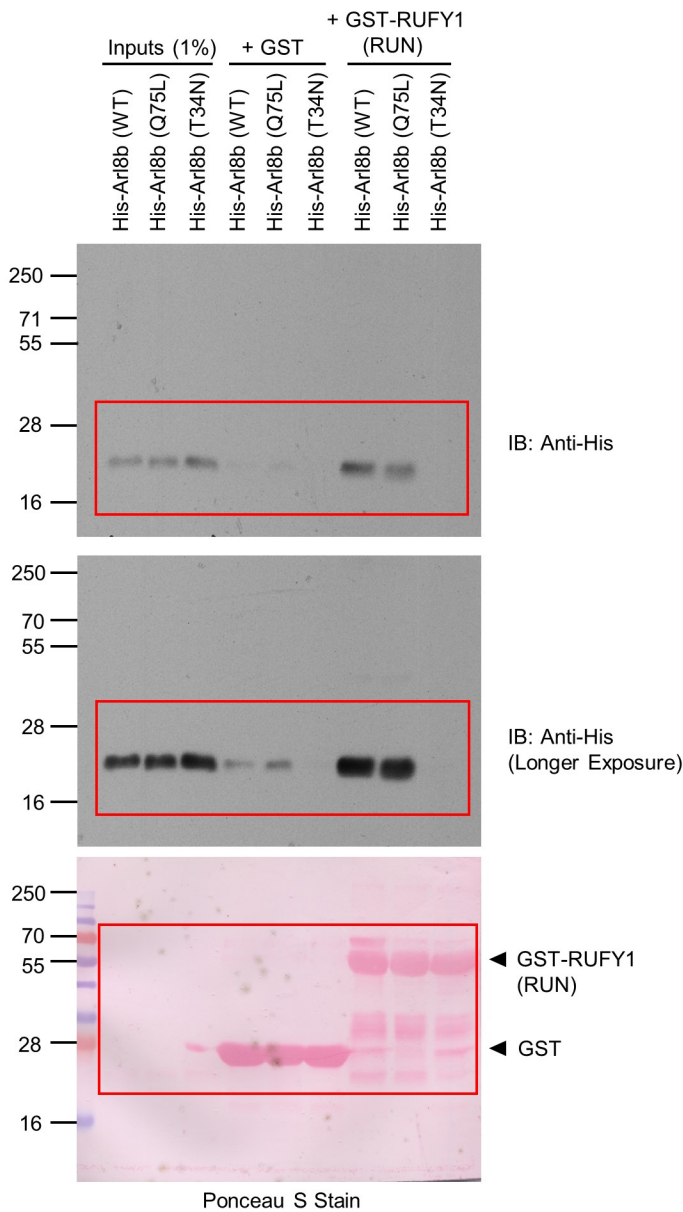

Supplement: SourceData F3 — is the source file for Fig. 3. [file JCB_202108001_SourceDataF3.pdf]

# Source Data Main Fig.4

Fig. 4E

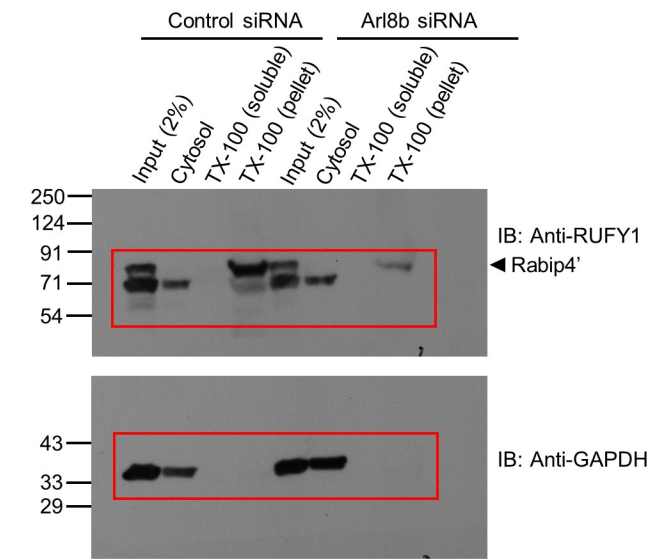

Fig. 4G

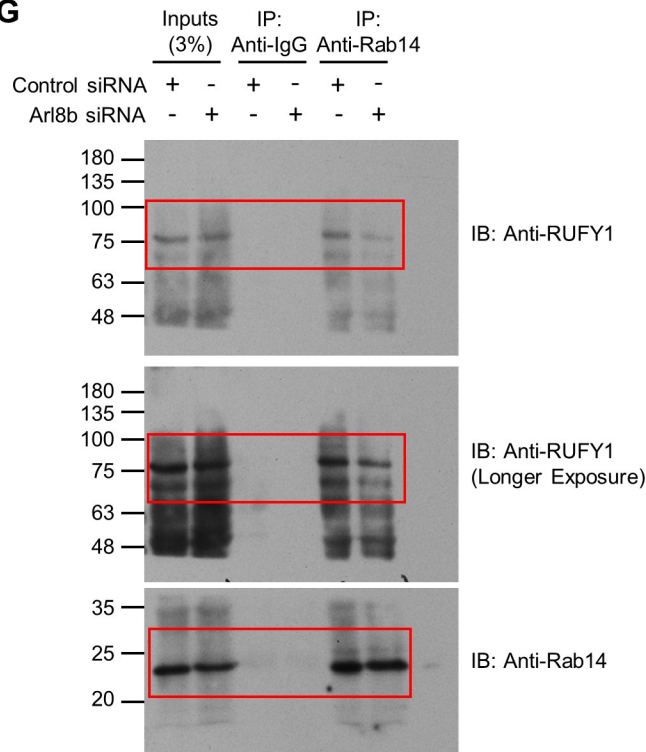

Fig. 4I

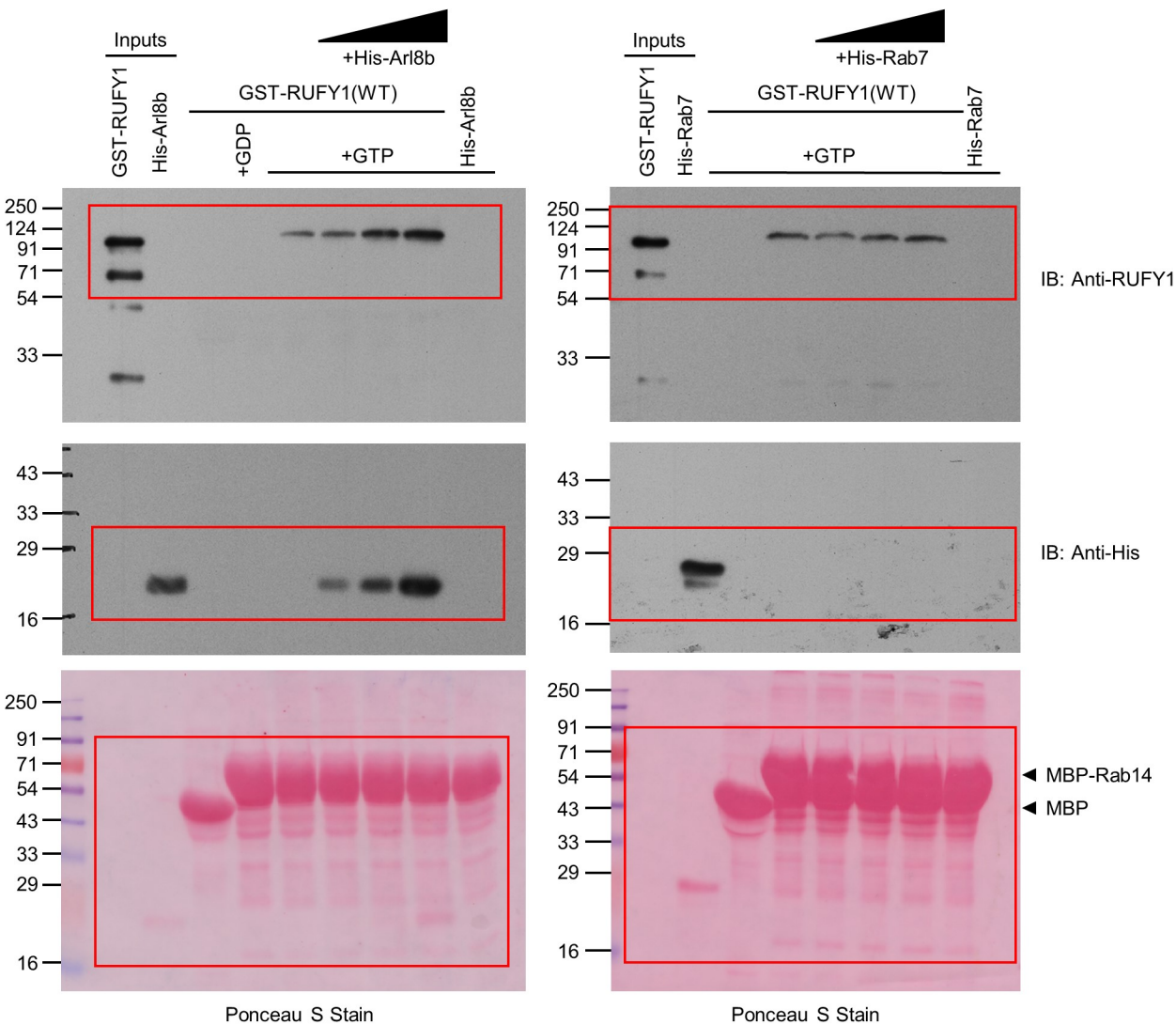

Supplement: SourceData F4 — is the source file for Fig. 4. [file JCB_202108001_SourceDataF4.pdf]

# Source Data Main Fig.7

Fig. 7K

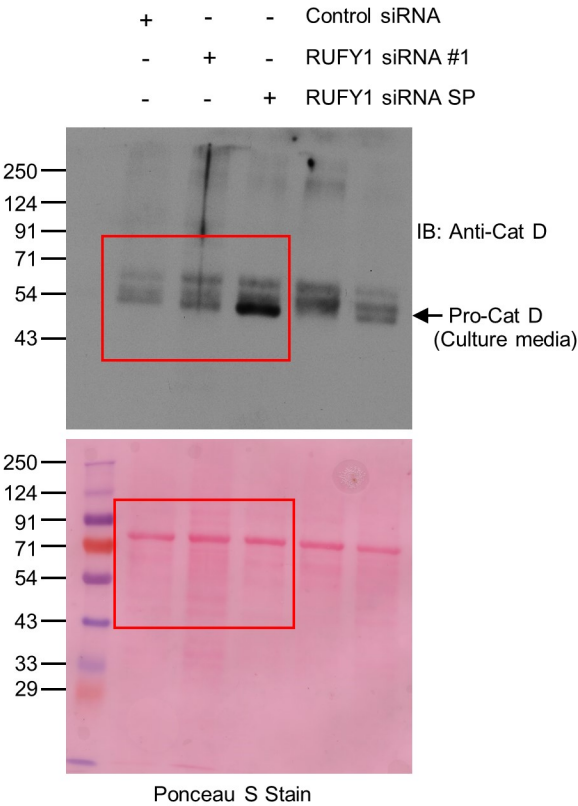

Supplement: SourceData F7 — is the source file for Fig. 7. [file JCB_202108001_SourceDataF7.pdf]

Source Data Main Fig.8

Fig. 8A

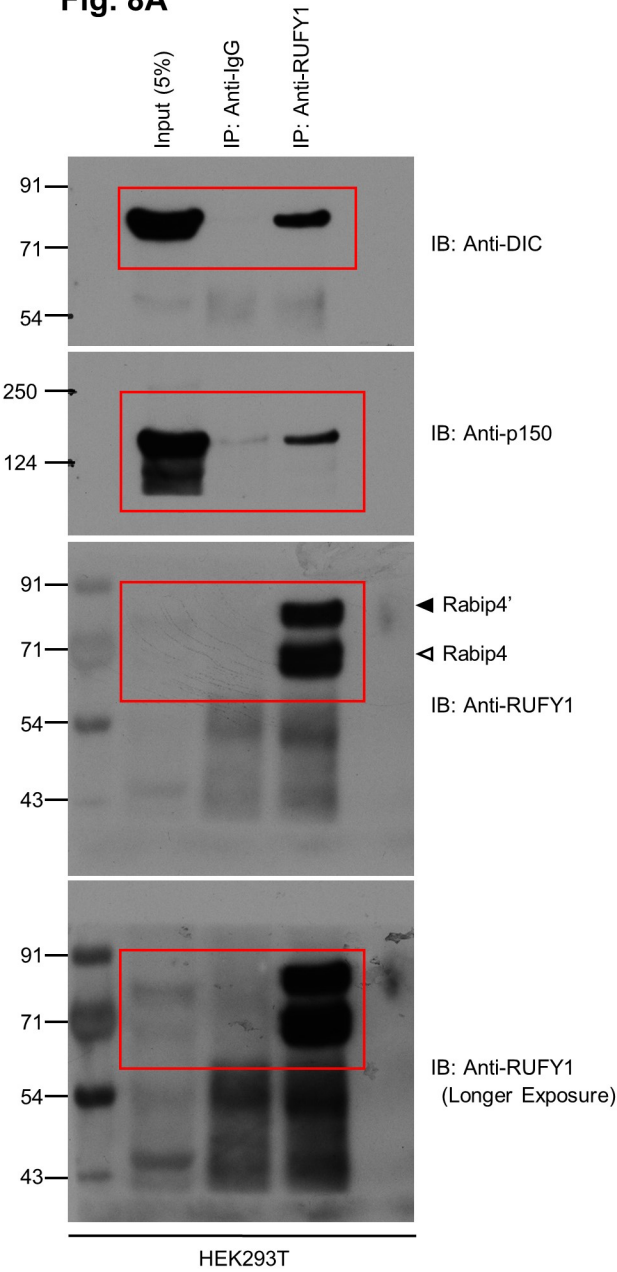

Fig. 8B

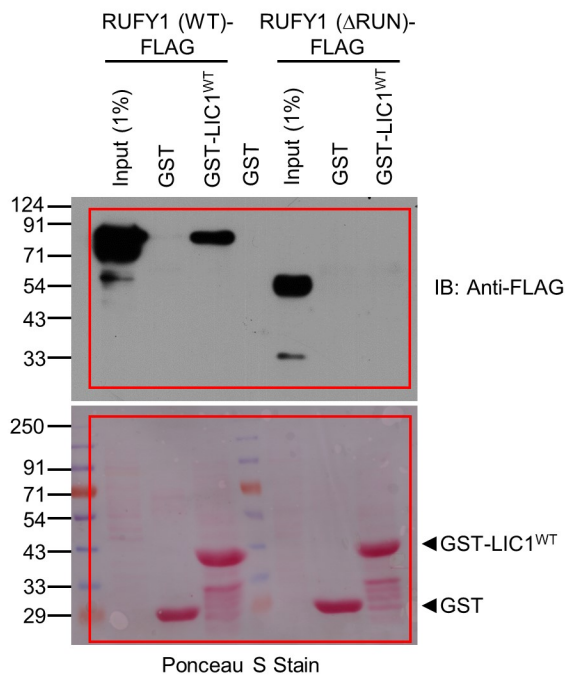

Fig. 8C

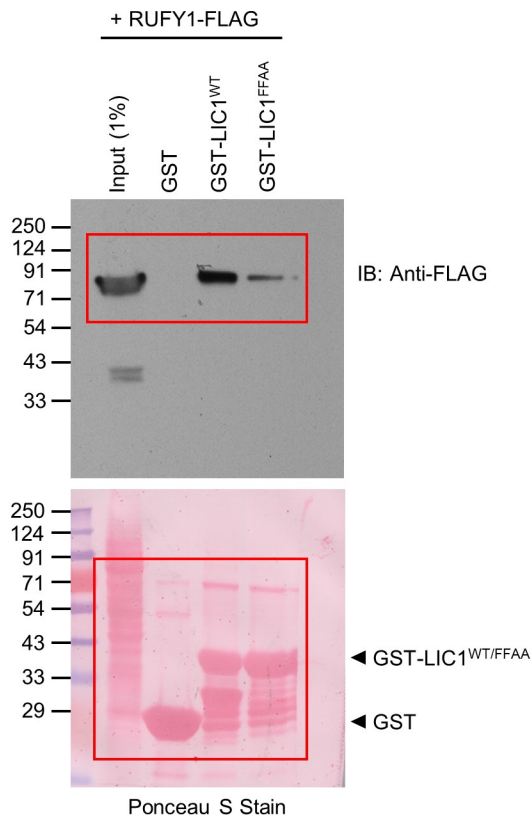

Supplement: SourceData F8 — is the source file for Fig. 8. [file JCB_202108001_SourceDataF8.pdf]

Fig. 9K

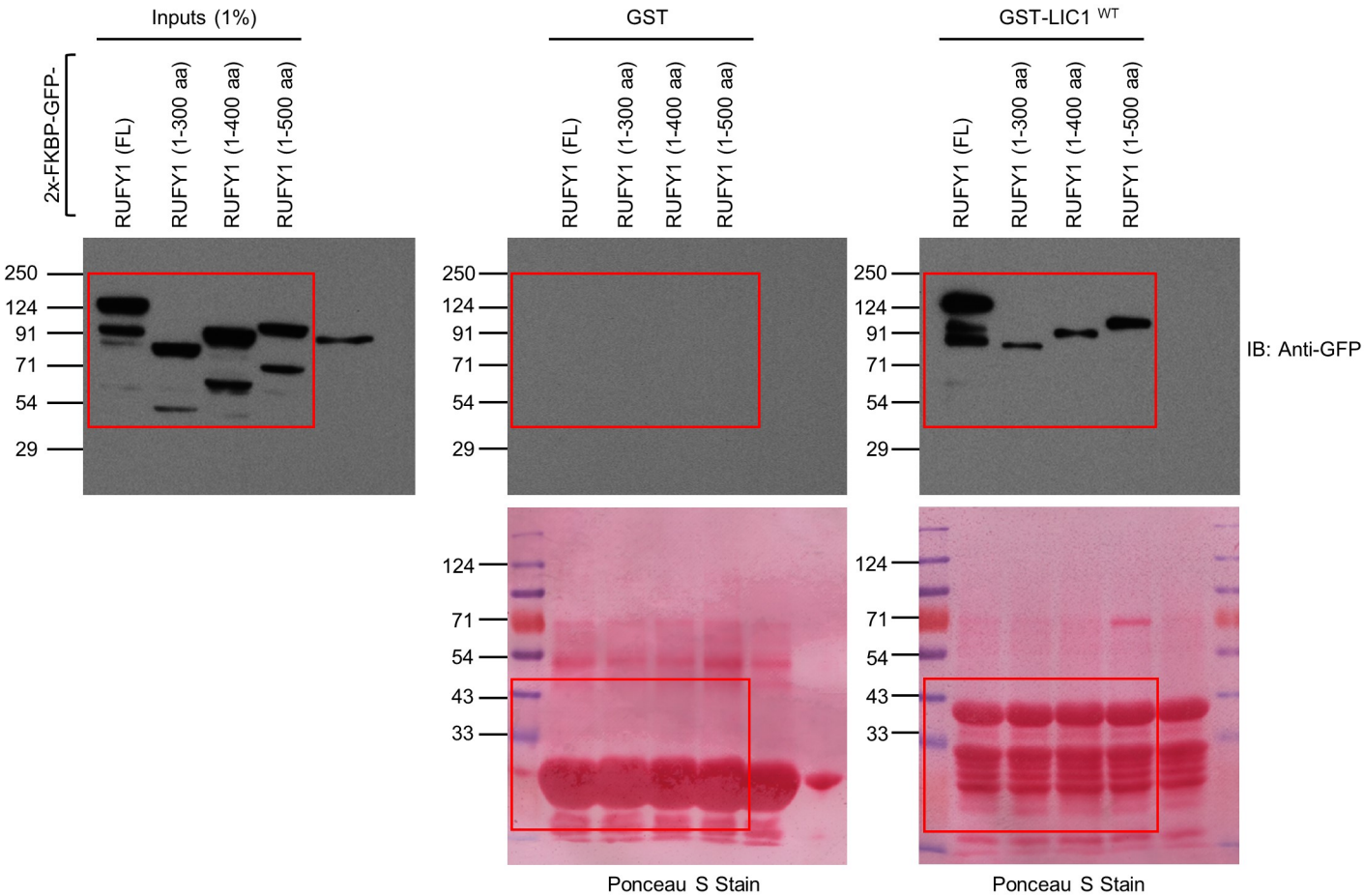

Fig. 9L

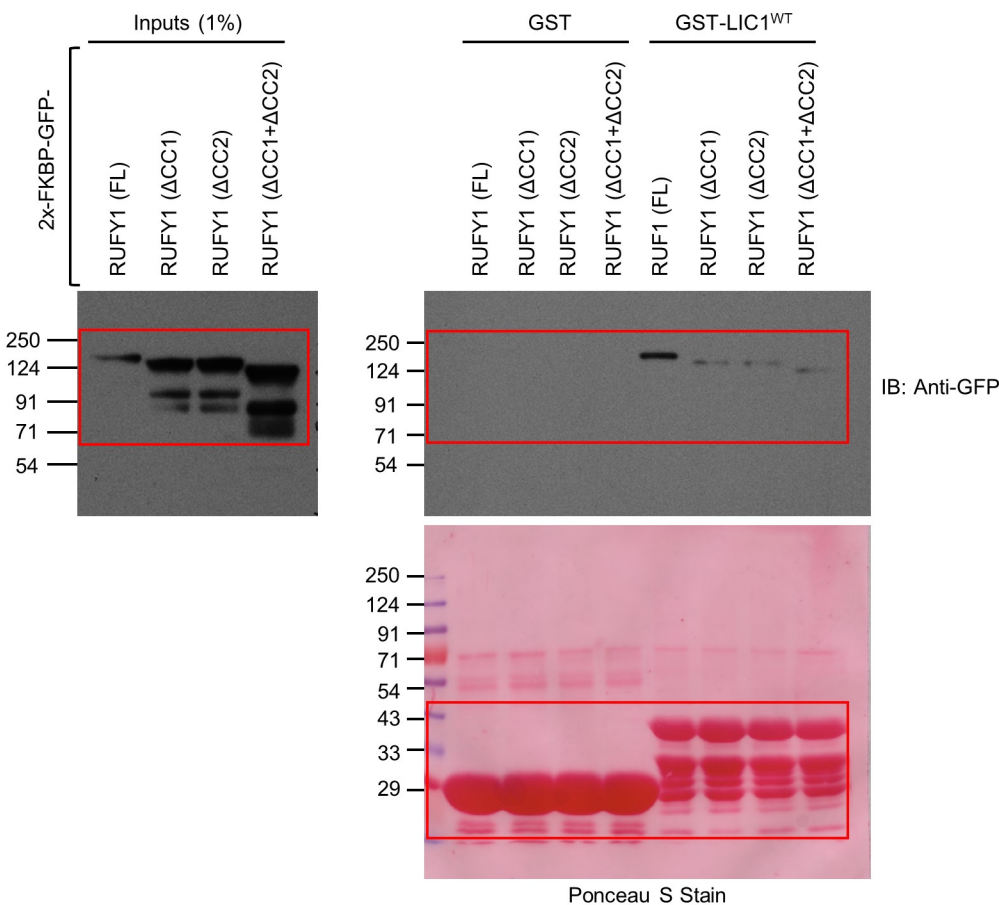

Supplement: SourceData F9 — is the source file for Fig. 9. [file JCB_202108001_SourceDataF9.pdf]

Source Data Supplementary Fig.S1

Fig. S1A

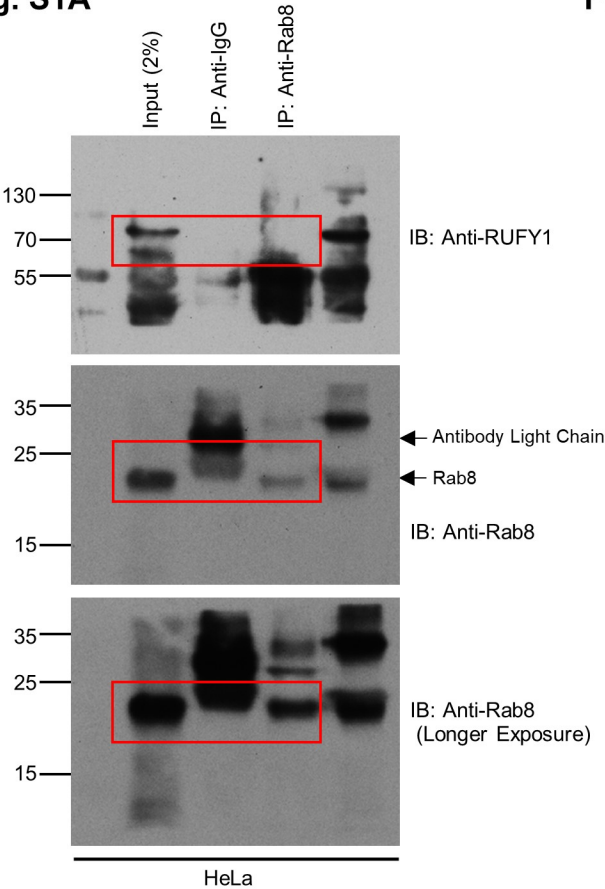

Fig. S1B

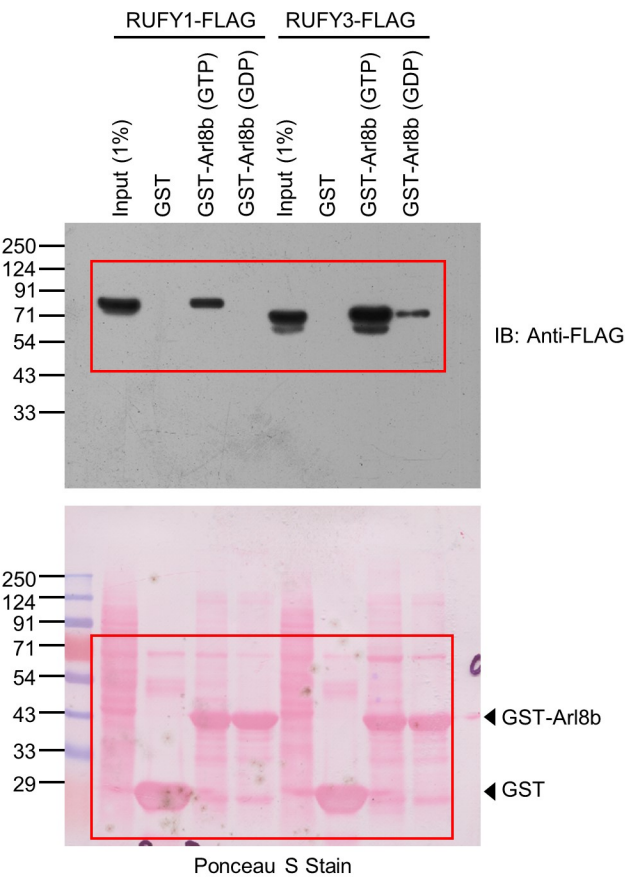

Supplement: SourceData FS1 — is the source file for Fig. S1. [file JCB_202108001_SourceDataFS1.pdf]

# Source Data Supplementary Fig.S2

Fig. S2F

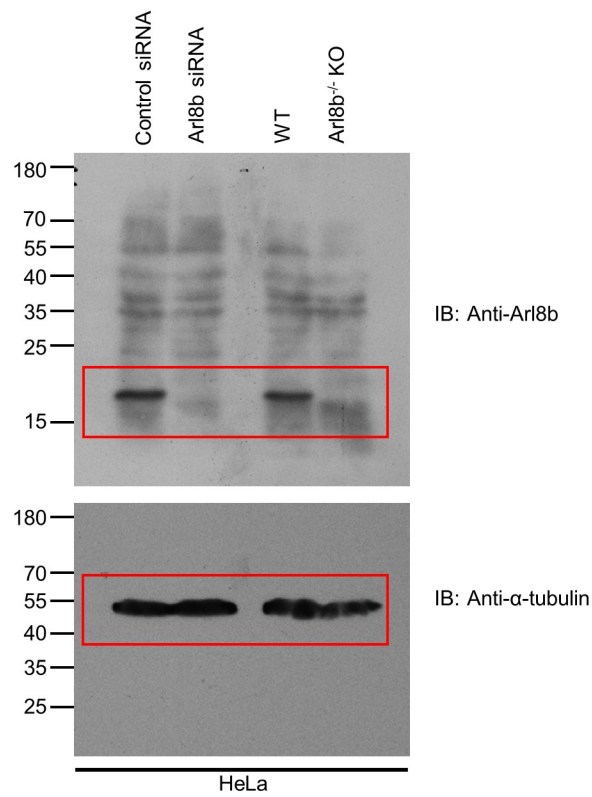

Fig. S2L

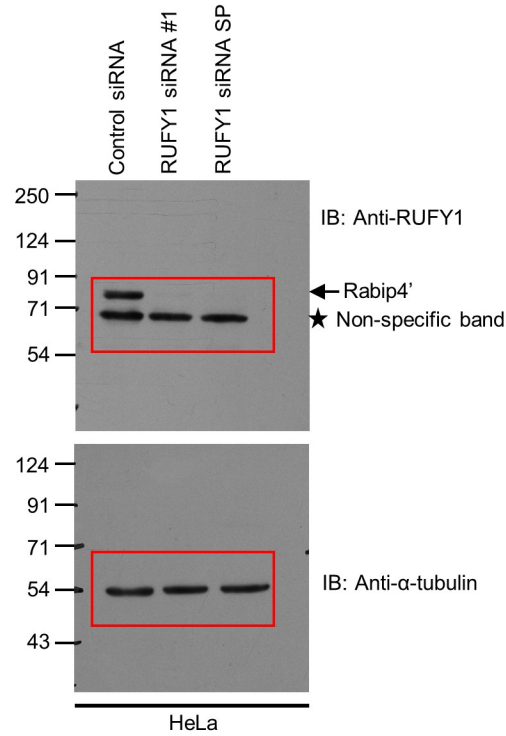

Supplement: SourceData FS2 — is the source file for Fig. S2. [file JCB_202108001_SourceDataFS2.pdf]

Fig. S3K

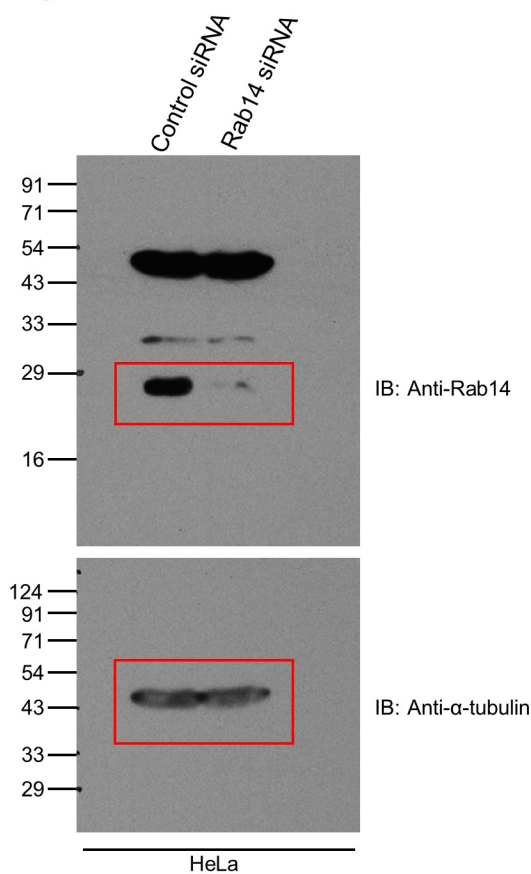

Supplement: SourceData FS3 — is the source file for Fig. S3. [file JCB_202108001_SourceDataFS3.pdf]

Fig. S4L

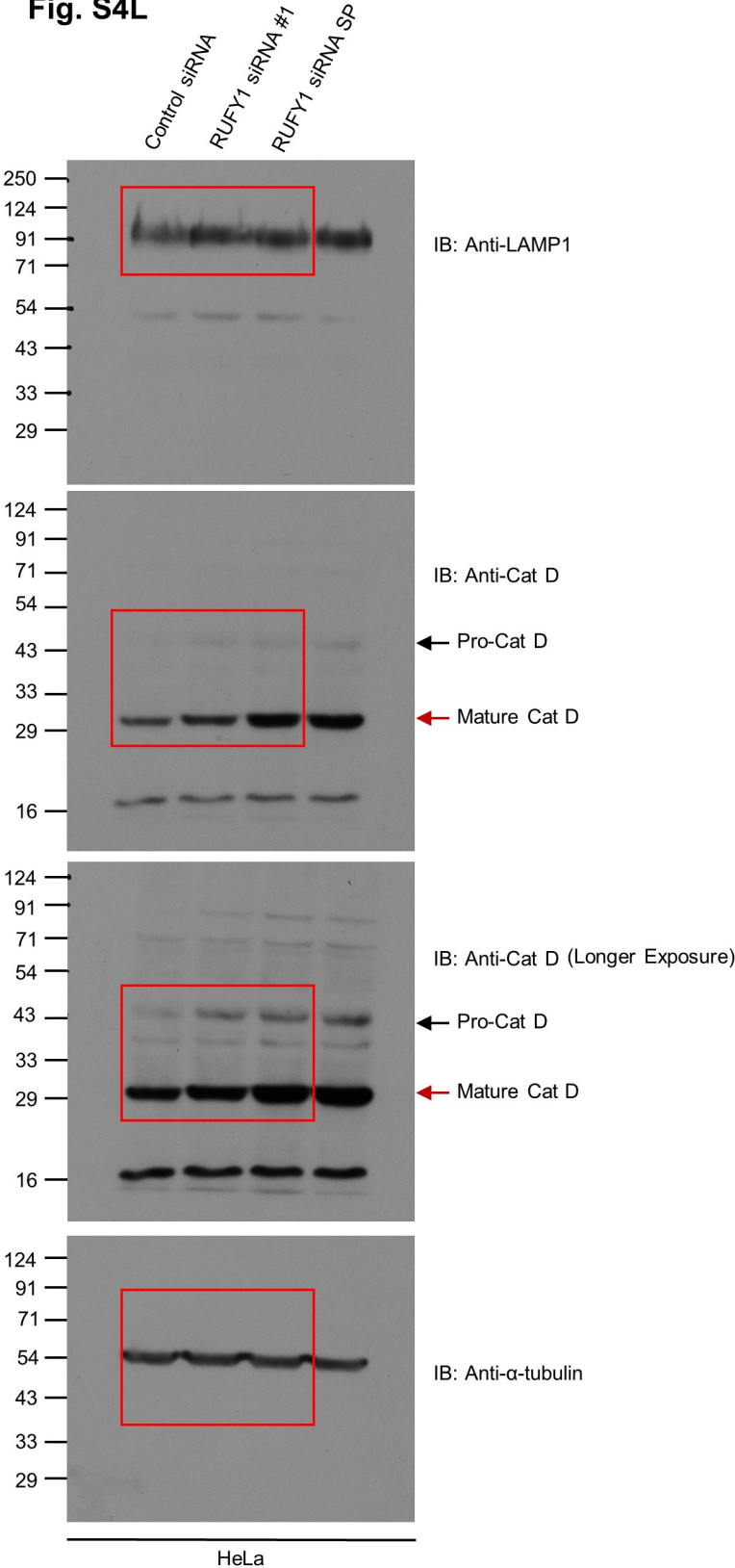

Supplement: SourceData FS4 — is the source file for Fig. S4. [file JCB_202108001_SourceDataFS4.pdf]

Fig. S5A

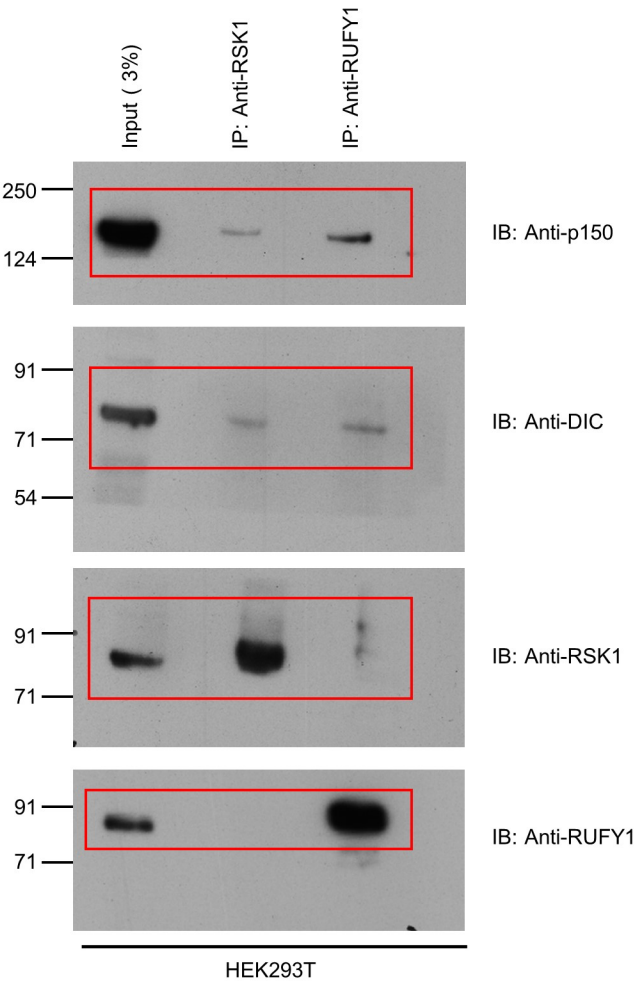

Supplement: SourceData FS5 — is the source file for Fig. S5. [file JCB_202108001_SourceDataFS5.pdf]
